# Regenerative agriculture augments bacterial community structure for a healthier soil and agriculture

**DOI:** 10.3389/fagro.2023.1134514

**Published:** 2023-05-05

**Authors:** Indira Singh, Meeran Hussain, G. Manjunath, Nagasuma Chandra, G. Ravikanth

**Affiliations:** 1 Ashoka Trust for Research in Ecology and the Environment, Bangalore, India; 2 Indian Institute of Science, Bangalore, India

**Keywords:** regenerative agriculture, conventional agriculture, soil microbiome, vegetable, ragi, soil organic carbon

## Abstract

**Introduction:**

Use of chemical fertilization and pesticides not only harm the environment but also have detrimental consequences on human health. In recent years, there has been a major emphasis worldwide on natural agriculture methods. Regenerative agriculture is known across the world as a combination of nature-friendly farming practices such as no-till, cover cropping, crop-rotation, agroforestry and use of organic home-based/farm-based ingredients to revive soil health. In India, a number of farmers are slowly adopting these practices using home-based mixtures and farmyard manure for soil rejuvenation and pest management. In order to evaluate the efficacy of the regenerative agriculture practices, this study compared conventional and regenerative agriculture plots for their soil bacterial and nutrient profiles.

**Methods:**

Two crops - ragi (Finger millet, an old world cereal eaten in India) and vegetable (tomato/beans), and different lengths (≤3 and >5 years) of regenerative practices were additional metrics considered to understand variabilities due to crop-type and period of application. The common regenerative agriculture practices used by farmers in this study included a mix of practices such as mulching, minimal-till, inter-cropping, crop-rotation, along with application of farmyard manure and other home-based concoctions rich in nutrients and microbes for enriching the soil.

**Results:**

We found that all regenerative practices were effective in bringing about an enrichment for soil bacteria with a more heterogeneous composition. Additionally, in regenerative vegetable (RV) versus conventional vegetable (CV) and barren land (BL) plots the relative percentage abundance of Actinobacteriota (RV-7.47%/ CV-6.24%/BL -7.02%) and Chloroflexi (RV-9.37%/ CV-6.63%/BL-8.75%) was slightly higher. In contrast, levels of Acidobacteriota (RV-8.1%/ CV-9.88%/BL-9.62%) was significantly lower. Similarly, regenerative ragi (RR) in comparison with conventional ragi (CR) and barren land (BL) plots saw higher representation of Firmicutes (RR-5.45%/ CR-2.38%/BL-1.45%) and Actinobacteriota (RR-11.53%/ CR-7.08%/BL-7.15%) and a concurrent reduction in Acidobacteriota (RR-6.91%/CR-7.39%/ BL-9.79%). The RV plots were found to be enriched for Plant Growth Promoting Rhizobacteria (PGPRs) - *Pseudomonas* sp. (RV-0.51%/CV-0.01%/BL-0.21%), and RR plots were enriched for *Bacillus* sp. (RR-1.35%/CR-0.95%/BL-0.61%), and *Mesorhizobium* sp. (0.30%/0.12%/0.21%), which are known to play significant roles in vegetable and ragi growth respectively.

**Discussion:**

Interestingly, long-term regenerative agriculture was able to support good nutrient composition while enhancing Soil Organic Carbon (SOC) levels. In all, the regenerative agriculture practices were found to be effective in improving bacterial community structure and simultaneously improving soil health. We found that BL soil with eucalyptus plantation showed among the least bacterial diversity suggesting detrimental impact on soil health.

## Introduction

Agriculture is the primary livelihood means for more than 50% of India’s population (Madhusudhan, 2015). With the advent of green revolution, farmers used conventional agriculture involving intensive use of synthetic fertilizers and pesticides for crop and field management (Prashar and Shah, 2016; John and Babu, 2021). Conventional agriculture with other unsustainable land management practices such as tilling, leaving the soil barren during non-growing season, agricultural intensification and monoculture cropping have led to the deterioration of soil quality and crop health, leaving the farmers economically distressed (Sathyanarayana Rao et al., 2017; John and Babu, 2021).

Regenerative agriculture, on the contrary, derives its roots from the traditional agriculture practices depending primarily on farm-based resources and methods, is environmentally conducive, climate-smart, healthier and holds prospects for sustainability in agriculture (Singh and Singh, 2017; Xiuli et al., 2018; Patel et al., 2020; Kim et al., 2021; Zhang et al., 2021). A healthy soil is supported by a robust and thriving microbial community, which can carry out a host of biogeochemical activities to enrich the soil with essential nutrients and plant growth promoters (Hartman and Tringe, 2019; Mahmud et al., 2021; Tripathi and Gaur, 2021). In this study, we compare two farming systems (regenerative and conventional) based on their soil nutrient and bacterial profiles to verify their abilities in restoring soil health in the context of Karnataka’s semi-arid farmlands.

Conventional agriculture, which involves application of chemical fertilizers (Nitrogen, Phosphorus and Potassium, NPK) for boosting agricultural outputs, has been implicated for acidification and deterioration of soil and climate change (Kumar et al., 2020). Excessive addition of nitrogen fertilizer brings about leaching of nitrogen into waterbodies, a major cause of eutrophication apart from accumulation and release of nitrous oxide from soil, a potent greenhouse gas. In contrast, regenerative agriculture uses environment friendly soil and crop management systems, which has the ability to heal the environment cost effectively with minimal inputs (Brodt et al., 2011; Peter et al., 2020; Schreefel et al., 2020; Giller et al., 2021).

Some of India’s smallholder farmers have recently started to adopt regenerative agriculture to improve their soil and crop health. Alongside using the globally practiced regenerative methods, smallholders in Karnataka also use soil-rejuvenation methods based on traditional knowledge. They use homemade additives made from cow-products and other easily available ingredients such as jaggery and chickpea flour for soil and crop-pest management. Although, there is a huge repertoire of knowledge accumulating to show the benefits of regenerative agricultural system, yet there is an ongoing debate on integrating the two systems to achieve sustainability in food production (Riaz et al., 2020; Srour et al., 2020). Consistent with this idea, many Indian farmers use both chemical fertilizers and farm-based manure for better yield (Pental, 2021; Aryal et al., 2021).

The soil microbial community is comprised of bacteria, fungi, viruses and protozoans. These microbes carry out the fundamental processes facilitating-nutrient cycling, decomposition of organic matter, defining soil texture, soil water-retention capacity, degradation of toxic wastes and preventing the growth of plant pests and pathogens (Brussaard et al., 2007). Different soil treatments can have an impact on the microbial community structure, but the microbiome changes are very complex processes stimulated by multiple factors such as temperature, climate, treatments, type of crop grown, cropping patterns, etc. Sustainable agriculture practices should ideally boost the growth and prevalence of beneficial microbes over the pathogenic species. Studies show that regenerative agriculture manifests soil health by improving soil microbial diversity and richness (Munnes and Mulugeta, 2014; Fierer et al., 2020; Bertola et al., 2021; Mulugeta, 2021). Indian farmers use a range of different regenerative agriculture practices including – mulching, cover cropping, inter and mixed cropping, no-till, use of soil and plant treatments such as farmyard manure, vermicompost, as well as using indigenous knowledge derived methods such as application of - Jeevamrutha (a fermented mixture of leaves, jaggery, chickpea flour, cow urine, cow dung, soil and water) as a pesticide, Beejamrutha (a seed-treatment mixture containing cow dung, cow urine, soil and lemon juice) and Panchagavya (a mixture of five cow-based ingredients – cow dung, cow urine, curd, milk and butter) (Patel et al., 2020; Duraivadivel et al., 2022). Some of the larger farms in India also use agroforestry for supporting the health of the farm and maintaining high productivity (Brown et al., 2018). In India’s Karnataka state - intercropping is widely used by the farmers by planting diversified crops including legumes (red gram, hyacinth beans, cowpea etc.), vegetable crops, millets (such as sorghum) and oilseeds such as caster simultaneously to improve agroecosystems and manage crop pests (Maitra et al., 2021). Study shows that no-till significantly affects the soil microbial diversity selecting for the beneficial microbes that improve nutrient translocation and protection of plant against pathogens while tilling leads to dominant accumulation of fungal saprotrophs and plant pathogens (Srour et al., 2020). Likewise, a comparative study of low-input/organic versus conventional farming found an enhancement of microbial diversity in organic agriculture soils, enriched for microbial guilds that can degrade manure and compost (Hartmann et al., 2015). Intercropping was found to improve soil Nitrogen (N) availability leading to increased plant N assimilation and improved grain size owing to changes in soil microbial structure (Dang et al., 2020). However, availability of too many regenerative agriculture options with little knowledge about their anticipated outcomes, followed by a long time-period for a demonstrable change in soil health/plant yield, makes a smallholder farmer desperate and vulnerable. Therefore, a scientific understanding of the basis of soil health promotion by these practices is essential for enabling an evidence-based recommendation. Additionally, due to availability of a broad range of regenerative practices, along with huge variabilities in regional soil types, climatic conditions, timing and extent of application and differences in crop type and cropping patterns, it is extremely difficult to compare studies from across the world. Therefore, a region specific and country specific study would be useful to obtain first-hand information on the mode of action and benefits accrued. To date there is no such study reported from India to show the comparative advantage of using regenerative agriculture on soil microbial diversity.

Metagenomics analysis using next generation high-throughput sequencing of soil DNA samples has been an efficient tool to determine the microbiome in soil. The technique provides details on the diversity, abundance and occurrence of specific genera and species in the given sample (Ding et al., 2015; Feng et al., 2018; Bertola et al., 2021; Ziqin et al., 2021; Smruthi et al., 2022). In the current study, using 16S metagenomics we compared using 16S metagenomics we compared the bacterial community structure under regenerative agriculture with that observed in conventional agriculture and barren land. Further, the metagenomics datasets were analyzed for alpha and beta diversity to establish the bacterial diversity in different samples. Agricultural plots growing either vegetable crops (tomato/bean) or finger-millet crop (Ragi) were considered here.

## Materials and methods

### Soil sample collection

This study aimed to establish the impact of regenerative agriculture practices on soil nutrient composition and microbial health with respect to the number of years of application. We considered two types of crops for this study – Ragi (finger-millet) and Tomato/Bean (Vegetable) crop primarily because these are the most commonly grown crops in this semi-arid terrain. Soil sampling was carried out in January and February of 2021 when there was a brief respite from Covid-19. Therefore, some samples were collected in absence of the crop. Soil was collected from near the roots of the crops wherever we could find plots with crops and for others soil was collected at the depth of 1-5 cm from the top. We collected soil from four corners of the plots and one from the center of the plot. Finally, all the soil samples from one plot were pooled together for experimentation. For chemical analysis, we collected about 2 kg of the soil pooling soil samples from all the five locations on the plot into one common bag. For the microbiome study soil was collected in sterile falcon tubes kept on ice and finally stored at -20 ^0^C until further processing. Soil sampling was done as given in Table 1.

We selected the plots for this study in the outskirts of Bengaluru in the towns of Ramanagara, Magadi, Doddaballapur and Hosur. This region is predominantly semi-arid. Barren land (BL) samples with no vegetation and with eucalyptus formed the no treatment controls. Barren land with eucalyptus (*BL-Euc*) was included as an additional metric in the study to get a sense of how monocultures impact soil health. The regenerative plots varied greatly in the kind of application practiced. For instance, some farmers used farmyard manure and Jeevamrutha, while others used farmyard manure, Jeevamrutha along with vermicompost (Table 1).

Sample grouping into categories for analysis:

- two conventional vegetable (CV) plots – *Con-VP1 & Con-VP2*
- two conventional ragi (CR) plots – *Con-RA1 & Con-RA2*
- two regenerative (≤3 years) vegetable (RV) plots – *Reg-1VA & Reg-3VP*
- three regenerative (>5 years) vegetable (RV) plots – *Reg-8VP, Reg-10VP & Reg-12VA*
- one regenerative (≤3 years) ragi (RR) plot– *Reg-1RA*
- two regenerative (> 5 years) ragi (RR) plots – *Reg-7RA* & *Reg-8RA*
- two barren land samples – *BL* (no vegetation) & *BL-Euc* (with Eucalyptus)

### Soil chemical analysis

Collected soil samples were taken to the laboratory, shade dried, pounded using wooden pestle and mortar, sieved (2 mm) and stored in airtight polyethylene bags for further analysis. The soil samples were analysed for various electrochemical properties. The soil pH, electrical conductivity, organic carbon content, nutrients namely - nitrogen, phosphorus, potassium, calcium, magnesium, and micronutrients - iron, zinc, manganese and copper were analyzed according to the standard procedures as given in Table 2.

### Soil DNA isolation, library preparation and deep sequencing

DNA was isolated from the soil samples using DNeasy Power soil kit, following manufacturer’s protocol. DNA samples were sent for 16S metagenomics analysis to Eurofins, where amplicon sequencing was done using Illumina MiSeq platform (Eurofins Genomics India Pvt. Ltd., Bangalore, India). The quality of the DNA samples was checked using NanoDrop estimation by determining A260/280 ratio. The amplicon libraries were prepared using Nextera XT Index Kit (Illumina inc.) as per the 16S Metagenomic Sequencing Library preparation protocol (Part # 15044223 Rev. B). Primers for the amplification of the bacterial 16S V3-V4 region were designed and synthesized at Eurofins Genomics Lab. Amplification of the 16S gene was carried out. The QC passed amplicons with the Illumina adaptor were amplified using i5 and i7 primers that add multiplexing index sequences as well as common adapters required for cluster generation (P5 and P7) as per the standard Illumina protocol. The amplicon libraries were purified by AMPure XP beads and quantified using Qubit Fluorometer. The amplified and AMPure XP bead purified libraries were analyzed on 4200 Tape Station system (Agilent Technologies) using D1000 Screen tape as per manufacturer’s instructions. After obtaining the mean peak sizes from Tape Station profile, libraries were loaded onto MiSeq at appropriate concentration (10-20 pM) for cluster generation and sequencing. Paired-end sequencing allows the template fragments to be sequenced in both the forward and reverse directions on MiSeq. Kit reagents were used for binding the samples to complementary adapter oligoes on paired-end flow cell. The adapters were designed to allow selective cleavage of the forward strands after re-synthesis of the reverse strand during sequencing. The copied reverse strand was then used to sequence from the opposite end of the fragment.

### Metagenomics analysis

In all, there were 14 samples and the number of read pairs ranged from 100,468 to 341,993 per sample. Quality check of 16S rRNA sequences was done using FastQC (v0.11.5) and the adapter sequences were removed using Trimgalore (version: 0.6.7) (Andrews, 2010; Andrews et al., 2015). The complete metagenome analysis was done using the QIIME 2.0 (Quantitative Insights Into Microbial Ecology) (version: 2021.4.0) pipeline (Bolyen et al., 2019). De-noising of the paired-end reads was done using the DADA2 tool that is within QIIME 2.0 which is used to filter low-quality reads of Phred score <15. High-quality reads were retained in 16S rRNA sequences by truncating the length of the forward read to 285 bp and the reverse reads to 250 bp. The resulting reads were de-noised to obtain unique sequence variants. DADA2 (version: 2021.4.0) produces “operational taxonomic units (OTUs)” by grouping unique sequences; these are 100% equivalent to the OTUs and are referred to as “Amplicon Sequence Variants (ASVs)”. The feature table was constructed using QIIME 2.0, which is similar to the BIOM table and the representative sequence file.

Further, the phylogenetic tree was built for each sample using the MAFT program, which is an inbuilt plugin in the QIIME 2.0 pipeline, results from this program are used to study the Alpha diversity by using Faith’s Phylogenetic and Pielou’s evenness matrix. Alpha diversity is further explored as a function of sampling depth by performing Alpha Rarefaction. Taxonomic classification was done by mapping the sequences at 99% sequence identity to an optimized version of the SILVA database using Naive Bayes classifier and q2-feature-classifier plugin of QIIME 2.0. The results of each step were downloaded from the QIIME2 program and they were plotted using ggplot2 (3.3.5) with R programming language (Wickham, 2016; Bolyen et al., 2019).

### Statistical analysis

Statistical analysis was performed using Welsh two sample unpaired t-test for comparing soil’s chemical properties and for comparing alpha diversity in different soil types. The Welsh two sample unpaired t-test was done using R-package (R Core Team, 2022). The comparison of phylum and genus level distributions was done using ANCOMBC method (Lin and Peddada, 2020). For differences with a p-value < 0.05 were considered significant.

## Results

### Soil’s organic carbon and nutrient composition

The chemical properties of soil such as - pH, major and minor nutrient composition obtained in the study were compared with pre-defined ideal values (given in Table 3). The results from the soil chemical analysis show that except *Con-RA2 (*pH = 3.94), *Con-RA1* (pH = 5.79), *BL* (pH = 5.85) and *BL-Euc* (pH = 6.04), all other samples had pH either in the ideal range (6.5-7.5) or in the moderately alkaline range (Rattan et al., 2012).

For most parameters, there was no significant difference between the conventional and regenerative agriculture plots. For instance, nitrogen levels were observed to be much less than the required range of 280 – 560 kg/ha in all the plots. Phosphorus levels were much above the required range of 22.9 – 56.3 kg/ha, while potassium was in the ideal range (141-3663 kg/ha) in all the soil samples. An important finding was that phosphorus and potassium are present at very high levels in *Reg-10VP* soil with the use of only organic manure. The *Reg-10VP* plot uses very heavy application of cattle manure and other household+ farm-based mixture and has been using these practices for as long as 10 years. It would be interesting to study how cattle manure and each of these practices individually contribute to soil’s phosphorus and potassium content. Additionally, *Reg-10VP* also showed the best organic carbon composition of 0.51% (ideal – 0.5 – 0.75%), unlike all other soil samples which remained below the ideal range. In contrast, the other regenerative agriculture plots in this study did not seem to show such a remarkable enhancement in their nutrient profiles when compared with the conventional agriculture soil. However, most regenerative plots have desired levels of most macro- and micronutrients barring nitrogen and organic carbon levels. This clearly indicates that most of these regenerative soil treatments regimens have the ability to provision maximum of these nutrients even in the absence of inorganic additives.

Further investigations will be needed to establish the basis for the improved chemical profiles in *Reg-10VP* soil. Altogether, these findings suggest that the long-term application of regenerative practices could help to improve the soil’s nutrient composition including organic carbon levels.

### Taxonomic composition of soil microbial community

To identify the bacterial community structure associated with conventional versus regenerative practices, we performed 16S metagenomics studies. The sequence data for all samples has been uploaded to the NCBI database and can be accessed at PRJNA912401. A total of 2,941,473 raw sequence reads from 14, 16S metagenome libraries were generated by the Illumina platform, ranging from 1,51,169 to 3,41,993 reads per sample. After removal of adapter sequences, ambiguous reads (reads with unknown nucleotides “N” larger than 5%), and low-quality sequences (reads with QV <20 phred score) and a minimum length of 100 bp, 2,801,991 high quality clean reads were further used for analysis.

The datasets were analyzed with QIIME 2.0 pipeline, using the SILVA database. At phylum level, Proteobacteria, Bacteroidota, Planctomycetota, Cyanobacteria, Actinobacteriota, Chloroflexi, Acidobacteriota, Verrucomicrobiota, Firmicutes and Gemmatimonadetes are the top 10 predominant phyla.

### Bacterial richness and community heterogeneity

Soil samples were classified into following groups for this analysis –

- Barren (comprising *BL* and *BL-Euc*);
- Conv (Vegetable plots-*Con-VP1* and *Con-VP2*) and (Ragi plots - *Con-RA1* and *Con-RA2*);
- Reg ≤ 3 (Vegetable plots - *Reg-1VA* and *Reg-3VP*) and (Ragi plots – *Reg-1RA*);
- Reg>5 (Vegetable plots – *Reg-8VP, Reg-10VP* and *Reg-12VA*) and (Ragi plots – *Reg-7RA* and *Reg-8RA*)

**Figure 1:**
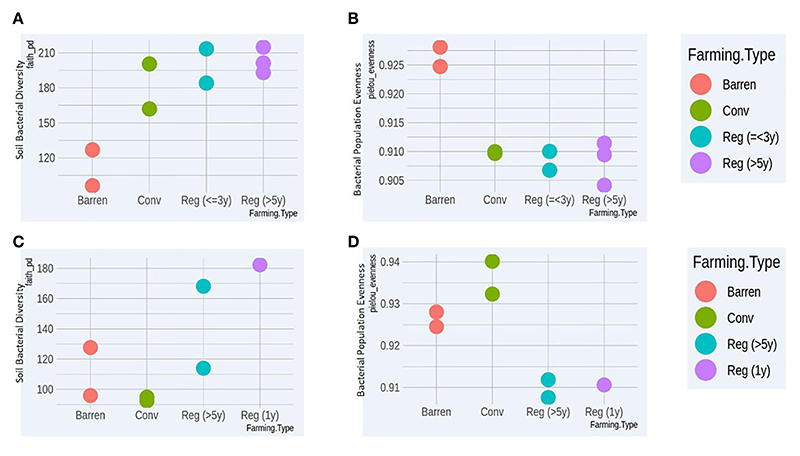
Comparative bacterial Richness **(A)** and Evenness **(B)** analysis of Vegetable growing conventional (*Conv*) and Regenerative agriculture (*Reg*) plots with Barren land (BL) soil. Comparative bacterial Richness **(C)** and Evenness **(D)** analysis of Ragi growing conventional (*Conv*) and Regenerative agriculture (*Reg*) plots with Barren land (BL) soil.

For both crop types (vegetable and ragi), we found that regenerative agriculture plots in general showed higher bacterial richness compared to conventional and barren (Figures 1A, C). Furthermore, bacterial species evenness comparison showed that both regenerative vegetable (RV) and regenerative ragi (RR) plots displayed least species evenness implying that the species composition in these plots is highly heterogeneous (Figures 1B, D). Surprisingly, CR plots showed least bacterial richness (Figure 1C) which was even less than that observed in the BL soil, whereas CV soil demonstrated better bacterial richness than BL samples (Figure 1A). On a similar note, CR plots had the highest species evenness followed by BL plots (Figure 1D), while CV plots had lower species evenness than BL (Figure 1B). Our findings indicate that regenerative agriculture increases soil’s bacterial richness and heterogeneity irrespective of crop type and the kind of regenerative practices adopted.

### Alpha diversity

The alpha diversity among different soil samples was compared to determine the mean species diversity in each plot. A higher alpha diversity value therefore signifies a more diverse pool of bacterial species accumulation. It is important to point out here that we collected a few soil samples from regenerative plots in the presence of vegetable crops labeled with the suffix V*P*, in the presence of ragi are labeled as *RP* and those taken post-harvest are labeled with the suffix V*A and RA* respectively. While all CV plot soils were collected in the presence of the crop, all CR plot soils were collected in the absence of the crop.

**Figure 2:**
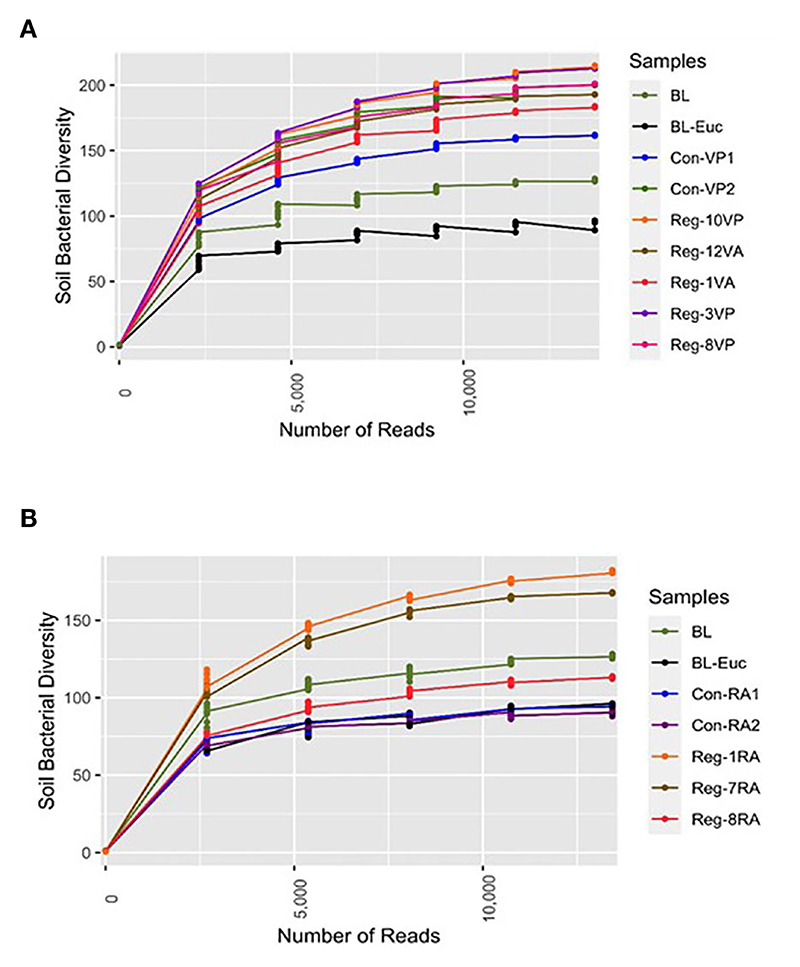
Alpha rarefaction study for soil bacterial diversity analysis of individual - **(A)** Vegetable growing Regenerative and Conventional plots with Barren land (BL). Significantly higher alpha diversity observed in RV versus BL soil; and **(B)** Ragi growing *Reg* and *Con* plots with BL. Significantly higher alpha diversity observed in RR versus CR soil; p<0.05 was considered significant.

Overall, the alpha diversity study showed that most regenerative agriculture plots demonstrated higher alpha diversity compared to conventional agriculture plots and barren soil (Figures 2A, B). Among vegetable plots our results indicate that alpha diversity is directly proportional to the length of regenerative agricultural practice. For example, the bacterial diversity in soil from vegetable regenerative plot practicing for 10 years (*Reg-10VP*) was greater than that observed for the plot practicing for 8 years (*Reg-8VP)* (Figure 2A). Likewise, among the post-harvest category, we observed greater bacterial diversity in *Reg-12VA* (12 years) as compared to *Reg-1VA* (1 year) (Figure 2A). Surprisingly, and in contrast to time-dependency, *Reg-3VP* (3 years) showed a better alpha diversity than *Reg-8VP* (8 years). We believe that this variability is due to the inherent differences in the soil quality associated with various locations. As expected, soil collected from RA plots where vegetable crops were present showed greater diversity than RA soil samples collected post-crop harvest (Figure 2A).

Another interesting observation was that *Con-VP2* soil, which is exposed to a combination of conventional and regenerative practices, displayed bacterial diversity comparable to that observed in *Reg-12VA* (Figure 2A). This result is significant as it shows that despite merging two agricultural methods and soil sampling done in presence of crop, yet *Con-VP2* had bacterial diversity only as good as *Reg-12VA* where soil was taken in the absence of crop. Thus, a definitive augmentation in soil bacterial speciation is observed in the plots selectively practicing regenerative agriculture.

In contrast to vegetable plots, soil from the ragi growing plots could only be collected post-harvest. It is noteworthy that the CR plots displayed as poor bacterial diversity as was found in *BL-Euc* (Figure 2B). Least bacterial diversity in these CR plots could be due to the degradative impact of conventional fertilization on the soil’s microbial health or due to continuous cultivation with no supportive interventions or due to the inherently poor soil quality of Doddaballapur from where these soils were obtained. Interestingly, while RR plots showed better bacterial diversity than CR, the duration of regenerative practices did not correlate with the bacterial species enrichment. For example, *Reg-1RA* (practicing for 1 year) displayed higher bacterial diversity than *Reg-7RA* (practicing for 7 years). Surprisingly, *Reg-8RA* (practicing for 8 years) displayed bacterial diversity lower than even the *BL* plot. One explanation could be that at different places the starting soil will have different baselines of bacterial diversity. The sample *Reg-1RA* was collected from Magadi while *Reg-7RA* and *Reg-8RA* were obtained from Ramanagara. It seems that Magadi soil is already healthier than soil from other places owing to its mostly green-covered scape and a more recent agricultural shift in the region compared to Ramanagara, Doddaballapur, and Hosur. Therefore, soil in other places demand higher inputs to be rejuvenated compared to Magadi soil. This argument is strengthened by the finding that *Reg-3VP* (Figure 2A) also coming from Magadi shows a bacterial profile as rich as that observed in *Reg-10VP* plot in just three years of regenerative agriculture practice.

### Bacterial community

**Figure 3:**
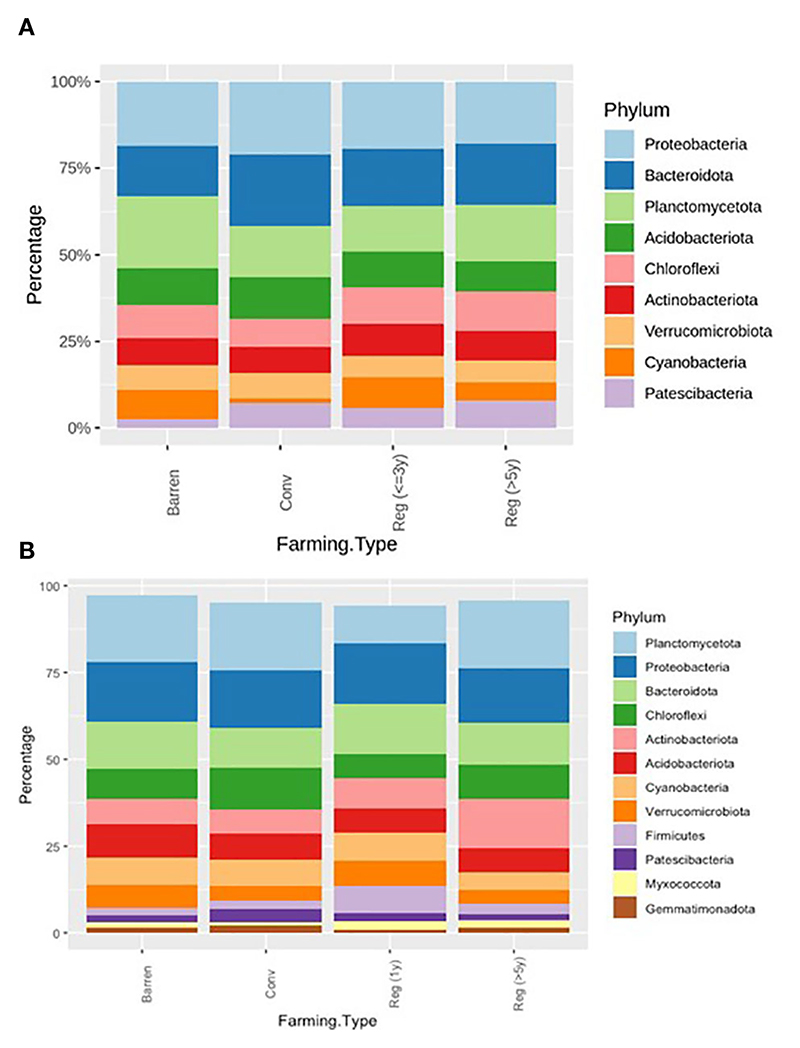
**(A)** Relative bacterial abundance at phylum levels in Conventional (*Conv*) and Regenerative (*Reg*) agriculture plots and BL in **(A)** Vegetable plots and in **(B)** Ragi plots. In RV versus CV – Cyanobacteria, Firmicutes and Gemmatimonadota had p < 0.05; In RV vs. BL – Proteobacteria, Planctomycetota, Chloroflexi and Gemmatimonadota had p < 0.05; In CV vs. BL – Proteobacteria, Planctomycetota, Acidobacteria, Chloroflexi and Cyanobacteria had p < 0.05; In RR vs CR – Myxococcota had p < 0.05.

To elucidate the bacterial community structure in the various types of plots, we assessed and compared the bacterial phyla associated with different soil samples grouped into categories as described previously in bacterial richness and heterogeneity analysis. The major phyla observed in both kinds of vegetable plots and Barren soil included – Proteobacteria, Bacteroidota, Planctomycetota, Acidobacteriota, Chloroflexi, Actinobacteriota, Verrucomicrobiota, Cyanobacteria and Patescibacteria (Figure 3A). Similarly, in ragi plots and barren soil comparison the bacterial community was majorly represented by the phyla – Planctomycetota, Proteobacteria, Bacteroidota, Chloroflexi, Actinobacteriota, Acidobacteriota, Cyanobacteria, Verrucomicrobiota, Firmicutes, Patescibacteria, Myxococcota and Gemmatimonadota (Figure 3B). Our observations show that in regenerative agriculture plots there is a shift towards a more uniform representation of all the major phyla compared to that in conventional agriculture plots. For instance, in vegetable plots (Figure 3A, Table 4A), we observed a reduction in the relative abundance of phyla Proteobacteriota and Acidobacteriota and an increased representation of– Chloroflexi, Actinobacteriota, Cyanobacteria and Patescibacteria in regenerative soil compared to conventional and barren soil. Similarly, in the ragi plots (Figure 3B, Table 4B) we observed relatively lower levels of Acidobacteriota and higher levels of Actinobacteriota and Fermicutes. Interestingly, a comparison to determine the impact of number of years of regenerative agriculture among RV plots did not show a significant change in the phylum level distribution in Reg ≤3 and Reg >5 soils. Although the comparison of RR plots (Reg >5 and Reg =1) (Figure 3B) showed a significantly higher representation of Firmicutes in Reg =1 soil despite only one year of regenerative practice. This is supposedly attributed to the regionally better soil of Magadi obtained Reg =1 soil (*Reg-1RA*). However, the RR plots practicing for Reg >5 years were found to show a significantly enhanced relative abundance of Actinobacteriota.

### PGPR community structure in regenerative agriculture

**Figure 4:**
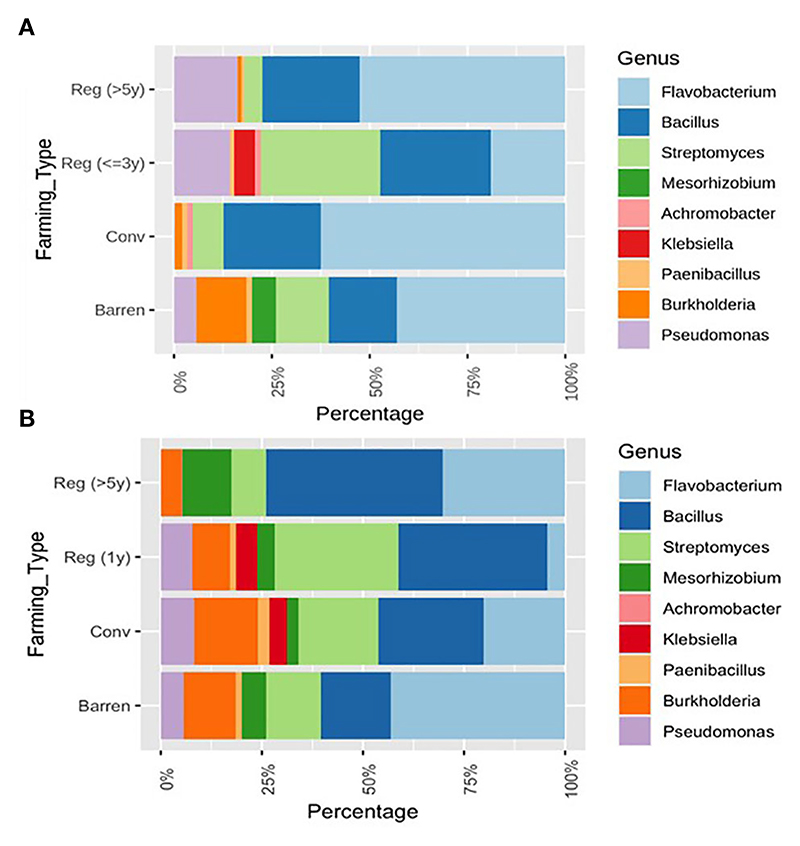
Relative composition of selected Plant Growth Promoting Rhizobacteria (PGPRs) in different soil samples. **(A)** Comparing Vegetable growing Regenerative (*Reg*) and Conventional (*Conv*) plots with BL and **(B)** Comparing ragi growing *Reg*. and *Conv*. plots with Barren. In CV vs. BL – *Klebsiella sps*. had p < 0.05; In RR vs. CR – *Streptomyces sps*. and *Burkholderia sps*. had p < 0.05; In RR vs. BL – *Burkholderia sps*. and in CR vs. BL – *Bacillus sps*. has p < 0.05.

Plant Growth Promoting Rhizobacteria (PGPR) are characterized to be an important group of soil bacteria that support plant growth and health by synthesizing and secreting various beneficial chemicals and nutrients in the soil. To determine the soil health in terms of PGPR representation, we selected a group of bacterial genera that have been well identified and classified as PGPRs (Beneduzi et al., 2012; Munees and Mulugeta., 2013; De Souza et al., 2015; Goswami et al., 2016; Backer et al., 2018). Among the genera considered here are – *Flavobacterium, Bacillus, Streptomyces, Mesorhizobium, Achromobacter, Klebsiella, Paenibacillus, Burkholderia* and *Pseudomonas*. Interestingly, RV plots when compared to CV and barren plot soils showed a relative enrichment for *Pseudomonas* sp. in the percentage ratio of - 0.51/ 0.007/0.21 for RV/CV/BL respectively. On the contrary, RR plots demonstrated an increased representation of - *Bacillus* sp. at - (1.35%/0.95%/0.61%) and *Mesorhizobium* sp. at (0.30%/0.12%/0.21%) for respective RR/CR/BL samples. The levels of *Bacillus* sp. are found to be significantly higher in both RR categories (Reg >5 and Reg = 1) compared to CR and barren land. The relative representation of *Mesorhizobium* sp. was found to be highest in Reg >5 in RR plots with a simultaneous reduction in levels of *Burkholderia* sp. compared to both CR and barren soil (Figure 4B). Interestingly, the genus *Streptomyces* was found to have a remarkably high representation in all Magadi plots (*Reg*-*1RA, Reg-1VA* and *Reg-3VP* compared to the other plots (Figures 4A, B). However, since we did not have any conventional plot or barren soil sample from Magadi it is impossible to estimate the contribution of RA on the enhanced *Streptomyces* configuration.

## Discussion

Regenerative agriculture has re-emerged in the last ten years (Giller et al., 2021) as a very important means of land rejuvenation practice for sustainability in soil health, farm productivity and environmental management. Regenerative agriculture provides us with a non-synthetic, nature-based option that helps to revive the ecosystem as a whole. In India too, there is growing interest in this environmentally-safe and less expensive agriculture system, necessitating the need for elucidating its impact on soil, environment and food production as a whole. Thus, this study has attempted to decipher the impact of regenerative agriculture on soil bacterial profile, soil nutrient composition, in two cropping systems under short (<=3 years) and long-term (>5 years) influence.

### Soil chemical properties

Most soil samples were found to have ideal pH or a somewhat alkaline pH, which is mostly suitable for agriculture. Acidic pH was found in the soil samples coming from Doddaballapur – *BL, BL-Euc, Con-RA1* and *Con-RA2*. These findings are consistent with reports showing that soil from Doddaballapur generally has an acidic pH in the range from 5.0 to 7.3 (Rajendra Hegde et al., 2007). Highest acidity in *Con*-*RA1* and *Con*-*RA2* soils are likely due to application of synthetic fertilizers and continued cultivation without allowing the land time to revive itself (Singh, 2018). As per the USDA, soils with pH below 5.5 are likely to have poor calcium, magnesium and phosphorus content (USDA, 1998). Consistent with this, *Con-RA2* with pH<5.5 and *Con-RA1* exhibiting pH around 5.5 showed low levels of calcium, magnesium and phosphorus. We further observed that soil samples with pH values above 7.8 have adequate calcium and magnesium levels but depleted copper, manganese and iron content. This was found to be somewhat true for the samples – *Reg-10VP* (pH = 7.95) and *Reg-8VP* (pH = 8.31) where calcium and magnesium levels are in surplus, whereas copper is much above the ideal limit of 0.2 ppm. Most regenerative agriculture plots were found to have ideal or slightly alkaline pH levels.

Available literature shows that as soil degrades there is a simultaneous decline in the composition of all its nutrients (Zhang et al., 2017). However, since the BL soils considered in this study did not show a marked reduction in any of the nutrients, therefore these soils may not be suitably classified as degraded. Although, it may be interesting to study the microbial health and nutrient composition of these soils in a span of 3-5 years from now, to observe the changes in the barren soil composition to estimate the progression of degradation.

### Bacterial richness and diversity

As shown by multiple studies from across the world, we found that regenerative agricultural system improves bacterial diversity compared to both conventional and barren soil (Harkes et al., 2019; Zheng et al., 2019; Kim et al., 2021; Mulugeta, 2021; Peltoniemi et al., 2021; Zhang et al., 2021). Here we report an increase in bacterial richness and heterogeneity across all regenerative plots, including those that have moved to this system very recently. This is a very significant result indicating that application of regenerative agriculture, from the outset boosts and modulates the soil’s bacterial growth, promoting a more heterogeneous composition for carrying out various soil health enhancing activities. Another important finding from the alpha diversity comparison of vegetable plots is that longer the period of RA application greater is the community’s bacterial diversity. These findings confirm the biological enrichment abilities of regenerative agriculture (Kumar et al., 2020; Peter et al., 2020).

The demonstrated lower alpha diversity among RA plots with no crops during soil sampling versus those with crops underpins the fact that roots of the crops induce proliferation of a large variety of root colonizing and plant growth stimulating rhizosphere microbes (Berg and Smalla, 2009; Benfey et al., 2010). Although the RR plots also showed the highest alpha diversity compared to CR and BL, yet a reverse time-dependence trend was observed among the ragi RA plots. This could be attributed to the inherent regional soil characteristics and composition that may be playing a significant role in shaping the microbial community structure (Zheng et al., 2019). This is evident from the Magadi obtained soils - *Reg-3VP* and *Reg-1RA*, which displayed highest alpha diversity in their respective groups despite the fewer years of regenerative application (Figures 2A, B).

Among all the RA plots in this study *Reg-10VP* was observed to show the best overall profile in terms of both bacterial community structure as well as soil chemical characteristics. Looking at the nutrient and bacterial profile of sample *Reg-10VP*, one can construe that continued regenerative practice over five years or more has the capability to improve the soil’s bacterial community structure, which would in turn enhance soil and plant health. We know from the farmer interviews that *Reg-10VP* has been demonstrating good crop yield. Furthermore, it is interesting to note that the Potassium, Phosphorus and Soil Organic Carbon (SOC) content of this soil is better than that of other farms. Studies have claimed that regenerative agriculture is the most promising way to sequester atmospheric carbon and mitigate climate change (Kell, 2012; Keith et al., 2019; Mattila et al., 2022). India’s soil is reported to be highly depleted in SOC levels (Bhattacharyya et al., 2009). A time series comparison of organic agriculture with conventional has shown that organic practice has helped improve SOC levels in soil from 12.5 g/dm^3^ to 21 g/dm^3^ and microbial biomass from 87 mg/kg to 120 mg/kg in a span of just one year (Araújo et al., 2009). An all-round improvement in soil bacterial and nutrient profile displayed by *Reg-10VP* holds a similar promise for regenerative agriculture in India.

The intermediate level of bacterial diversity in CV plots is most likely due to the mixed agriculture methods used by these farmers. Here the farmers integrate both organic manure and chemical fertilization methods to accrue the benefits from both the systems. If used judiciously, the synthetic fertilizers may also be useful to supplement the soil with necessary nutrients and in maintaining the soil’s organic matter (SOM) (Pental, 2021; Singh, 2018; Giller et al., 2021). BL soil’s poor bacterial richness and high evenness is attributed to absence of any vegetation for multiple years resulting in continued exposure to weathering, erosion and deterioration (Qiu et al., 2021). Studies conducted on degraded soil in China reveal that poor quality soils display a depleted Operational Taxonomic Unit (OTU) richness for beneficial microbes and significant enhancement of pathogenic microbes (Zhang et al., 2017).

### Bacterial community structure

In RV plots we observed an increased representation of Chloroflexi, and Actinobacteriota. Actinobacteriota have been suggested to induce plant root biomass and thus supporting better nutrient acquisition (Yadav and Yadav., 2019; Ha et al., 2021; Hannula et al., 2021). Role of Chloroflexi in plant health is not clear although study has reported that Chloroflexi comprising anaerobic bacteria, are found to be enriched in paddy fields depending on oxygen availability and regulate soil bacterial community composition (Tang et al., 2021).

Likewise, the RR plots showed an enrichment for Firmicutes and Actinobacteriota population, which again form a group of extremely beneficial plant growth promoting bacteria (Yadav and Yadav., 2019; Hashmi et al., 2020). Phylum Firmicutes comprises a number of agro-ecologically beneficial bacterial genera, such as *Bacillus, Paenibacillus, Lysinibacillus, Brevibacillus, Planococcus, Clostridium, Sporosarcina* etc (Hashmi et al., 2020). Many of these bacterial genera (eg. *Bacillus*) have been identified as biocontrol and phytoremediation agents and others as Plant Growth Promoting Rhizobacteria (PGPRs). Thus, enrichment for Firmicutes in regenerative agriculture plots signifies a marked improvement in soil health. Members of the phylum Actinobacteriota like *Streptomyces, Brevibacteria* and *Nocardia* promote plant growth as bio-fertilizers and bio-controllers for agricultural sustainability (Yadav and Yadav, 2019). Similarly, a study has also shown the significance of both Firmicutes and Actinobacteriota in controlling bacterial disease incidence in tomato plants (Lee et al., 2021).

Barren soil was observed to have a relatively higher representation of Planctomycetota compared to both conventional and regenerative soils. Additionally, we observed a higher level of phylum Acidobacteriota representation in barren soil when compared with CR and RR plots. This is in coherence with a report where an increase in relative abundance of Proteobacteria, Acidobacteriota and Bacteroidota was observed in degraded soils whereas healthy soils were enriched for Actinobacteriota and Firmicutes (Zhang et al., 2017).

### Plant growth promoting Rhizobacteria

New developments in the field have shown that healthy soils are enriched in Plant Growth Promoting Rhizobacteria (PGPRs). These PGPRs secrete plant growth hormones and regulatory chemicals in the rhizosphere, facilitating plant growth by enabling plant nutrient procurement, modulating plant hormone levels and by releasing biocontrol agents to protect plants against pathogens. Many bacterial genera including *Pseudomonas, Bacillus, Streptomyces, Flavobacterium, Achromobacter, Mesorhizobium, Paenibacillus, Sinorhizobium, Burkholderia, Rhizobium*, etc. have been classified as PGPRs. Many of these bacteria are being currently used as biocontrol agents and as bio-fertilizers (Goswami et al., 2016; Pathak et al., 2018; Qessaoui et al., 2018; Riaz et al., 2020; El-Sersawy et al., 2021; Mekonnen and Kibret., 2021; Pirttilä et al., 2021). Augmentation of these bacterial genera in soil directly indicate towards improvement in soil health.

Our study showed a relative enrichment for *Pseudomonas* sp., in RV plots, *Bacillus* sp., and *Mesorhizobium* sp. in RR plots. Many studies have provided evidence that *Pseudomonas* forms the core of PGPRs for many vegetable, fruit and flowering plants (Qessaoui et al., 2018; Mekonnen and Kibret, 2021). According to studies, *Pseudomonas* is the most efficient producer of ammonia and enhances bioavailability and bio-assimilation of nutrients, promoting plant growth and yield (Qessaoui et al., 2018). Thus, enrichment for *Pseudomonas* sp. is essentially a favorable development in RV plots. Interestingly, studies show that ragi growth is promoted by the rhizospheric growth of *Bacillus* sp. The *Bacillus* sp. support ragi growth by fixing nitrogen and protecting the crop against the foot-rot disease causing pathogen, *Sclerotium rolfsii* (Choudhary et al., 2020). Furthermore, *Bacillus* sp. are known to be involved in improving the nutritive value of the ragi grains by enriching them with essential amino acids (Dheeman et al., 2020). An Ethiopian study suggests that *Bacillus* and *Pseudomonas* species form significant PGPRs supporting vegetable crops (Mekonnen and Kibret, 2021). In effect an enrichment for *Pseudomonas* sp. in RV plots and for *Bacillus* sp. in ragi plots signify a beneficial transformation in soil bacterial composition. Likewise, *Mesorhizobium* sp. are found to be very useful PGPRs with their special property of synthesizing ACC deaminase enzyme which protects plant against abiotic stress by degrading ACC which forms the precursor for ethylene. Additionally, *Mesorhizobium* sp. synthesizes IAA which promotes plant root growth and also is involved in inorganic phosphate solubilization making it available to plants (Muleta et al., 2021).

Magadi soil seems to be inherently enriched in *Streptomyces* sp. *Streptomyces* sp. also form an important group of agriculturally beneficial rhizosphere bacteria (Manullang and Chuang, 2020; Srinivas et al., 2020). *Streptomyces* synthesize plant hormone – Indole acetic acid (IAA) in moderate quantities and help in phosphate solubilization and stress tolerance thus boosting plant growth and productivity. Thus this clearly indicates that regenerative agriculture practices are able to induce a healthy microbial population in the soil for promoting soil’s overall health and agricultural productivity.

### Regenerative practices and their impacts

Almost all regenerative agricultural plots considered here have indicated to the use of farmyard manure as an important supplement for soil management. Manure addition has been ascribed to inducing increased microbial biomass in soil (Das et al., 2017; Faissal et al., 2017; Semenov et al., 2021). Some studies indicate that the type and source of farm manure dictates the soil microbial population (Jiao et al., 2021). However, it may be difficult to define the source of origin of a microbe in soil. For instance, one report claims that cow manure enriches the soil for Firmicutes and Bacteroidota while another suggests an enrichment for Firmicutes and Proteobacteria. Contrary to this, a recent study claims that in a span of two weeks from manure addition, the microbes coming from the manure are mostly lost while the soil-borne microbes are activated to grow and multiply (Semenov et al., 2021). Regenerative plots demonstrated an increased growth of Firmicutes particularly *Bacillus* sp. in ragi fields and Proteobacteria (*Pseudomonas* sp.) in vegetable plots. In addition, since almost all the regenerative farms are using multiple regenerative practices apart from just farmyard manure application, these additional treatments will also influence the soil microbiome. More studies are therefore required to ascertain the roles of these individual treatments in determining the microbial community structure. In *Reg-10VP* plot a rich supplementation of farmyard manure (400 kg/row) could have been a significant contributing factor to the plot’s best nutrient and bacterial profile. However, since not all farms will be able to afford this kind of soil supplementation regimes, policies and practices such as encouragement of circular economy to provide household based compost to farmers is necessary.

### Influence of region and crop on soil bacterial composition

Soil microbial community structure was found to be influenced by regional and spatial characteristics. Certain regions required greater inputs with many years of application and others much less to achieve a credible improvement in microbial health and soil quality. This is evident from the Magadi obtained soil samples – *Reg-1VA, Reg-3VP* and *Reg-1RA*. These regenerative agriculture plots have been practicing for just one, three and one year respectively, yet these soils showed very high alpha diversity (Figures 2A, B) and a distinctly heterogeneous and highly diverse bacterial composition with a higher representation of *Streptomyces* sp. (Figure 4A, B). Additionally, type of crop also plays a role in defining the soil’s bacterial community structure as is evident from the varied profiles exhibited by regenerative plots growing ragi and vegetable crops (Jacoby et al., 2017; Xiuli et al., 2018; Hartman and Tringe, 2019).

### Soil microbiome impacts of merging conventional and regenerative systems

The *Con-VP2* where soil sampling was done in presence of crop forms a suitable example of a plot where the two agricultural systems – Conventional and Regenerative have been integrated for land and crop management. This plot shows a distinctly high alpha diversity comparable to that in *Reg-12VA* plot, where soil was collected in absence of crop. However, the alpha diversity of *Con-VP2* is still found to be lesser than all the RV plots where soil was taken in the presence of the crop. Thus, here we conclude that addition of synthetic fertilizer may have an adverse impact on the soil microbiome. Too much dependence on inorganic fertilizers comes with a host of adverse effects in soil including increase in salinity, acidification, soil compacting and poor water retention, impact on biogeochemical processes by altering microbial dynamics, accumulation of toxic wastes/heavy metals and finally reduced microbial diversity (Prashar and Shah, 2016).

### Limitation

This study was started in January 2021 when India was witnessing few Covid-19 cases. However, by March 2021 the pandemic raged throughout the country leading to very strict implementation of lockdown measures by the government, making it impossible for second and third round of soil sampling. By the time things settled down it was November 2021 when the soil profile had changed due to multiple contributing factors (e.g. weather, new round of cropping, etc.). Hence, the findings of this study are from one round of soil samples and could not accommodate for the required statistical comparison between multiple replicates of soil samples.

## Conclusion

This study aimed to compare and elucidate the effectiveness of regenerative agriculture practice on soil microbial and nutritive health with respect to conventional agriculture and barren soil. Barring a few exceptions owed to different original baselines of the selected plots, the observations show that extended periods of regenerative practice does improve soil bacterial diversity and soil nutrient health. Even SOC levels were found to be within the desired range in long-term regenerative application plots.

The RA plot showing the best bacterial profile and ideal SOC levels uses very heavy application of farmyard manure for soil management and Jeevamrutha for pest management. Thus although regenerative agriculture has the ability to induce beneficial outcomes in soil health and agriculture, the required impact is made possible only with a heavy use of amendments at least in the initial decade or so. This identifies the need for instituting a continued and surplus supply of manure to the farmers for ensuring high grade outputs.

## Figures and Tables

**Table 1:** Soil sampling, classification, agricultural practices and history.

Type of Crop	Sample Names	Place of Soil Sample Collection and Latitude/Longitude / Altitude (in m)	Soil Classification (as per USDA soil taxonomy)	Type of Agriculture	Previous Crop	No of Plots	No of Years of Practice	Major Agriculture Practices	History of the plot
Vegetable (Beans/Tomato)	*Con-VP1* and *Con-VP2*	Ramanagara *Con-VP1* *-*12.785677/77.218218/747 m *Con-VP2* - 12.785252/77.217067/747 m	Fine, Mixed, Isohyperthermic, Kandic Paleustalfs	Conventional	*Con*-*VP1* - Crop rotation with leafy vegetables. *Con*-*VP2* – crop rotation with carrots	2	–	Use of NPK fertilizers and chemical pesticides along with farmyard manure	Both *Con-VP1* and *ConVP2* have been following mixed agriculture practice by using both faryard manure and chemical treatment to support soil and plant health for 5-10 years. The adjacent plots use regenerative practices and intercropping and agroforestry etc which would also impact these plots.
	*Reg-* *1VA*	Magadi 12.956508/77.190900/925 m	Clayey-skeletal, Mixed, Isohyperthermic, Kandic Paleustalfs	Regenerative	None as the land was fallow since last ten years. This was the first crop	1	1	Cow dung, vermi-compost, and Jeevamrutha, crop rotation and inter-cropping; Seed treatment with Beejamrutha. Since this was the first crop they did not do crop residue management, slight till of 3-6 inches was done.	The plot was a Paddy and Ragi grown land a long time ago but no farming was being done on this plot since 2018. The land was acquired by the current farmer in 2020. He prepared it for doing agriculture and followed the practices as given in adjacent column. This was the first crop that this farmer had grown.
	*Reg-* *3VP*	Magadi 13.031895/77.326871/925 m	Clayey-skeletal, Mixed, Isohyperthermic, Kandic Paleustalfs	Regenerative	Green leafy vegetables	1	3	Farm manure and Jeevamrutha applied twice a year and mixed-cropping and crop rotation and agroforestry Neem oil for pest control. Seed treatment with *Pseudomonas* and *Trichoderma*	Farm plot was uncultivated for 20 years. For the past 3 years regenerative agriculture was being practiced as given in adjacent column. Farmer has been growing vegetables and fruit trees from past five years
	*Reg-* *8VP*	Ramanagara 12.783899/77.216949/747 m	Fine, Mixed, Isohyperthermic, Kandic Paleustalfs	Regenerative	Chilli	1	8	Farm manure, and Jeevamrutha applied twice a year, mixed cropping, crop rotation and Beejamrutha. 3-6 inch minimal tillage and using rotovator for managing crop residue and then it is added with farm manure followed by mulching	This farmer has been consistently practicing regenerative agriculture as discussed in adjacent column for 8 years. Before that for 12 years he was doing inter-cropping growing caster, red gram, hyacinth beans, cowpea and sorghum.
	*Reg-* *10VP*	Hosur 12.609869/77.702348/880 m	Loamy, Mixed, Isohyperthermic, Typic Rhodustalfs	Regenerative	Horse Gram	1	10	400 kg Farm manure per bed twice a year and Jeevamrutha through drip and spray, mulching of crop residue and Panchgavya. Crop rotation with legumes. For some seeds *Pseudomonas* treatment was given*	This large farm was previously used for cultivation of vegetable crops for 3 years by natural methods. For the past ten years since the land was acquired by the current owners, regenerative agriculture has been practiced here as given in adjacent column.
	*Reg-* *12VA*	Ramanagara 12.785475/77.219237/747 m	Fine, Mixed, Isohyperthermic, Kandic Paleustalfs	Regenerative	*Sesbania* *bispinosa*	1	10 -12	4-5 tons’ green leaf manure, farmyard (cattle) and Vermi compost, per year, Jeevamrutha and microbial culture added monthly twice during crop growth; inter-cropping and crop rotation with *Sesbania bispinosa*; seed treated with Beejamrutha and cow urine. Crop residue is managed with rotovator and eventually used as green manure. Minimal till of 3-6 inches.	Previously the land was a conventional chemical agriculture land. For past 12 years the farmer adopted regenerative agriculture. He has been growing multiple varieties of ragi, rice etc. He has developed a seed-bank. Now he grows multiple varieties of vegetables and greens and fruits using regenerative agriculture methods as outlined in adjacent column
Ragi	*Con-RA1 &* *Con-RA2*	Doddaballapur *Con-RA1*-13.377086/77.543199/880 m *Con-RA2*-13.374351/77.549439/880 m	Fine, Mixed, Isohyperthermic, Typic Haplustepts	Conventional	*Con*-*RA1* - The plot was a mango plantation before the current ragi crop Con-RA2 - Ragi with mixed crop as red gram, cowpea, caster, sorghum, hyacinth beans etc.	2	–	Use of NPK fertilizers and chemical pesticides alone. No other supplementation	The plots have been doing ragi cultivation for the last 3 – 5 years using conventional methods. Another disadvantage for these plots is their vicinity to eucalyptus plantations in their neighboring plots.
	*Reg-* *1RA*	Magadi 12.998967/77.306946/925 m	Clayey-skeletal, Mixed, Isohyperthermic, Kandic Paleustalfs	Regenerative	Ragi with mixed crop as red gram, cowpea, caster, sorghum and hyacinth beans etc are grown year after year	1	1	Farm manure and green manure, mulching; natural insecticide for pest management. This was first year no crop residue or mulching done. Minimal till of 3-6 inches.	It was an abandoned land for a long time until the current farmer acquired the land and started the cultivation using regenerative agriculture methods as given in adjacent column
	*Reg-* *7RA*	Ramanagara 12.779501/77.269773/747 m	Fine, Mixed, Isohyperthermic, Kandic Paleustalfs		Horse gram	1	7	cow dung, jaggery, Vermi-compost developed from crop residue, Jeevamrutha; A special organic pesticide + cow urine spray for pest management; crop rotation with legumes, crop rotation; seed treatment with Beejamrutha, cow dung, jaggery and calcium for seed treatment; organic pest management	Initially the farmer grew mango and ragi the conventional way. Later he also tried to grow mulberry but that failed. For the past seven years he has been practicing regenerative agriculture as detailed in adjacent column
	*Reg-* *8RA*	Ramanagara Regenerative	Fine, Mixed, Isohyperthermic, Kandic Paleustalfs	12.783881/77.217184/747 m	Ragi with Mixed crop such as red gram, cowpea, caster, sorghum and hyacinth beans etc. grown year after year	1	8	Farm manure, green leaves manure and Jeevamrutha applied twice a year; mixed cropping, seed treatment with cow urine; pest management also with cow urine and natural pesticide	This farmer has been consistently practicing regenerative agriculture as discussed in adjacent column for 8 years. Before that for 12 years he was doing inter-cropping growing caster, red gram, hyacinth beans, cowpea and sorghum.
Barren Land	*BL-Euc* *& BL*	Doddaballapur Not applicable	Fine, Mixed, Isohyperthermic, Typic Haplustepts	13.376992/77.542568/880 m	Not applicable	2	–	No treatment	The land has either been lying barren for 20 years or more or planted with eucalyptus

In the provided names the following nomenclature has been followed – Reg, Regenerative; Con, Conventional; BL, Barren Land. V, Vegetable; R, Ragi; P - soil sampling in Presence of crop; and A - soil sampling in Absence of the crop; and the numbers after the hyphen indicate the number of years of Regenerative agriculture practice. Jeevamrutha – composed of soil, chickpea flour, jaggery, cow dung and cow urine; Panchagavya – composed of milk, butter, curd, cow dung and cow urine; Beejamrutha – comprises of cow dung, cow urine, soil and lemon juice.

**Table 2:** Methods adopted for soil analysis.

Sl. No.	Parameter	Method
1.	Soil reaction (pH) (1:2.5 soil: water suspension)	Potentiometry (Kondvilkar et al., 2017)
2.	Electrical conductivity (1:2.5 soil: water suspension)	Conductometry (Kondvilkar et al., 2017)
3.	Organic carbon (%)	Wet oxidation (Walkley and Black, 1934)
4.	Available Nitrogen (kg ha^-1^)	Macro kjeldahl Distillation (Subbiah and Asija, 1956)
5.	Available Phosphorus (kg ha^-1^)	Spectrophotometry (Olsen et al., 1954)
6.	Available Potassium (kg ha^-1^)	Flame photometry (Kondvilkar et al., 2017)
7.	Exchangeable Calcium and Magnesium (mEq/1000 g)	Complexometric titration (Kondvilkar et al., 2017)
8.	DTPA extractable Iron, Manganese, Zinc and Copper (ppm)	Atomic Absorption Spectrophotometry (Lindsay and Norwell, 1978)

**Table 3:** Chemical parameters of the soil samples.

Sample Name	pH	EC (dS/ m)	Organic carbon (%)	Nitrogen	Phosphorus	Potassium	Calcium	Magnesium	Zinc	Manganese	Iron	Copper
				kg/ha			mEq/1000 g		ppm			
IDEAL	6.5- 7.5	<1.00	0.5-0.75	280-560	22.9-56.33	141-336	>1.5	>1.0	>0.6	>2.0	2.5 - 4.5	>0.2
*Con-VP1*	7.54	0.367	0.39	131.8	342.47	250.3	38	21	3.72	7.2	35.64	1.17
Con-VP2	7.6	0.399	0.44	106.6	346.28	284.4	41	27	4.77	7.68	11.31	0.84
*Reg-1VA*	7.41	0.113	0.29	125.4	87.64	217.9	35	19	1.23	6.33	13.32	0.42
*Reg-3VP*	7.43	0.193	0.3	156.8	62.39	184	40	30	4.44	3.99	32.16	1.56
*Reg-8VP*	8.31	0.267	0.36	120.1	152.8	189.7	62	47	2.34	6.27	8.79	0.72
*Reg-10VP*	7.95	0.279	0.51	144.2	510.13	506.4	105	64	4.08	8.34	19.62	2.19
*Reg-12VA*	7.71	0.231	0.32	100.3	187.33	223.5	79	54	2.79	9.42	16.53	0.81
*Con-RA1*	5.79	0.316	0.35	131	37.6	334	14	6	1.32	10.8	16.56	0.45
*Con-RA2*	3.94	0.159	0.39	144	44.7	170	21	10	1.14	18.63	78.27	1.5
*Reg-1RA*	7.35	0.128	0.38	106.6	58.58	216.6	58	32	1.05	11.01	10.32	0.51
*Reg-7RA*	6.89	0.09	0.36	119.1	148.61	252.7	45	28	2.58	14.58	36.18	1.56
*Reg-8RA*	7.01	0.13	0.42	125.4	60.01	306.7	54	38	2.37	16.02	23.13	0.57
*BL-* *Euc*	6.04	0.235	0.31	119	29	242	27	14	1.51	23.91	9.3	0.459
*BL*	5.85	0.106	0.41	150	14.7	108	22	9	0.99	6.33	16.29	0.327

The ideal values are based on recommendations given by the Indian Society of Soil Science (Rattan et al., 2012). pH and Phosphorus showed p<0.05 in comparison of RV vs BL, CV vs. BL; Calcium showed p<0.05 in RV vs. BL, CV vs. BL, RR vs. BL and RR vs. CR; Magnesium showed p<0.05 in RV vs BL, CV vs BL, RV vs CV, RR vs BL and RR vs CR; Zinc showed p<0.05 in RV vs BL and in CV vs BL; Copper was significantly different in RV vs BL. Rest differences were non-significant.

**Table 4A:** Percentage abundance of selected phyla chosen based on their relative abundance observed in vegetable and ragi plots.

Source of Soil	Proteobacteriota	Bacteroidota	Planctomycetota	Acidobacteriota	Chloroflexi	Actinobacteriota	Verrucomicrobiota	Cyanobacteria	Patescibacteria	Fermicutes
Regenerative Vegetable (<=3 years)	16.62	13.98	11.16	8.78	9.11	7.80	5.22	7.72	4.81	3.23
Regenerative Vegetable (>5 years)	15.26	14.83	13.85	7.42	9.64	7.15	5.39	4.47	6.73	2.83
Conventional Vegetable	17.45	16.95	12.30	9.88	6.63	6.25	6.02	1.14	5.96	2.12
Barren	16.88	13.07	18.90	9.62	8.75	7.02	6.47	7.70	2.31	1.86

**Table 4B:** Percentage abundance of selected phyla chosen based on their relative abundance observed in ragi plots.

Source of Soil	Planctomycetota	Proteobacteriota	Bacteroidota	Chloroflexi	Actinobacteriota	Acidobacteriota	Cyanobacteria	Verrucomicrobiota	Fermicutes	Patescibacteria
Regenerative (<=3 years)	10.87	17.55	14.32	6.88	8.94	6.81	8.17	7.16	8.01	2.32
Regenerative (>5 years)	19.66	15.79	11.81	10.05	14.12	7.02	5.07	4.07	2.89	1.78
Conventional	19.67	16.63	11.31	11.93	7.08	7.39	7.56	4.41	2.38	3.70
Barren	19.25	17.19	13.3	8.92	7.15	9.79	7.85	6.59	1.90	2.35

## Data Availability

The datasets presented in this study can be found in online repositories. The names of the repository/repositories and accession number(s) can be found below: https://www.ncbi.nlm.nih.gov/, PRJNA912401.
